# Influence of phenolic substrates utilised by yeast *Trichosporon cutaneum* on the degradation kinetics

**DOI:** 10.1080/13102818.2014.901671

**Published:** 2014-06-04

**Authors:** Maria Gerginova, Plamena Zlateva, Nadejda Peneva, Zlatka Alexieva

**Affiliations:** ^a^ Bulgarian Academy of Sciences, Institute of Microbiology, Sofia, Bulgaria; ^b^ Bulgarian Academy of Sciences, Institute of System Engineering and Robotics, Sofia, Bulgaria

**Keywords:** phenol, phenol derivatives, biodegradation, yeast, kinetic parameters

## Abstract

The degradation kinetics of different phenolic substrates utilised by *Trichosporon cutaneum* R57 was studied. The following compounds were used as substrates: *phenol*, resorcinol, hydroquinone, 3-nitrophenol, 2,6-dinitrophenol, 3-chloro phenol and *p*-cresol. The specific degradation rates (*Q_s_*) were described by a Haldane kinetic model. The unknown model parameters were estimated using the mathematical optimisation procedure for direct search. The results obtained demonstrated that *Q_s_* varied greatly in the experiments carried out. The level of biodegradability depended on the different structure and toxicity of compounds used as carbon substrates. The highest *Q_s_* values were observed for less toxic hydroxylated phenols (0.77–0.85 h^−1^), while the most toxic chlorinated phenols were characterised with the lowest *Q_s_* values (0.224 h^−1^). The results obtained with different concentrations of resorcinol (from 0.2 to 0.8 g L^−1^) and 2,6-dinitrophenol (from 0.2 to 0.7 g L^−1^) demonstrated a growing inhibitory effect directly correlating with the extended time necessary for complete degradation of both compounds.

## Introduction

The development of new advanced technologies for clean environment is directly dependent on the discovery of new evidence of the ability of living nature to remove harmful chemical pollutants that destroy it. Microorganisms play a crucial role in these processes. Data on the detection, characterisation and conditions of technological application of active microbial destructors as well as in improving the discovery of new environmental products and techniques are the result of the efforts of many different scientists, engineers and other professionals in environmental protection.

Most biodegradation processes are a result of the ability of microbes to accomplish complete utilisation of toxic pollutants of a chemical nature. Aromatic compounds are widespread in nature as a result of their involvement in many processes of chemical, food, textile and other industries and, hence, their entry into the wastewater. The catabolic activity of microorganisms might vary significantly depending on the chemical structure of the used substrates. The Environmental Protection Agency of the USA has classified phenol derivates as a pollutant of group C (possible human carcinogens) (http://www.epa.gov/waterscience/ criteria/wqcriteria.html).

The metabolism of aromatic compounds, particularly phenol and its derivatives, has been intensely studied with bacteria.[1–3] A very large amount of data have been gathered on bacterial species of genus *Pseudomonas*, which are known for their ability to use various aromatic compounds as a single source of carbon and energy.[4–7]

Several yeast species belonging to different genera: *Candida*, *Rhodotorula* and *Trichosporon*, which can biodegrade aromatic compounds have been described.[8–10] Along with the previously described representatives of *Candida tropicalis*, some other strains have also been reported because of their significant potential to degrade high concentrations of phenol.[11–13] The yeast strain *Trichosporon cutaneum* R57 is distinguished as an effective biodegrader able to utilise and thus remove a number of toxic aromatic compounds from the environment.[14–18]

The microbial degradation dynamics of toxic or inhibitory carbon sources is of considerable interest. Many data should be gathered and processed to determine the course of degradation in regard to predictive modelling of such processes.[19–21] Several reports compare the ability of different types of mathematical models to precisely describe the dynamics of such biodegradation processes. Most of them emphasise on the ability of Haldane's model to best describe this type of inhibitory kinetics.[22] The substrate inhibition kinetics studied in the process of phenol degradation by a strain of *Pseudomonas fluorescence* was fitted to Haldane, Yano and Koga, Aiba et al., Teissier and Webb models. Among the five inhibition models, the Haldane model was found to give the best fit.[18,23] Nevertheless, some authors prefer to use different models depending on the microbial strain and conditions of cultivation and utilisation of toxic substrates.[24]

The present study deals with the processes of degradation and utilisation of phenol and its monohydroxyl derivatives (resorcinol and hydroquinone), as well as some of the most toxic phenolic pollutants of the environment like 2,6-dinitrophenol, 3-nitrophenol, 3-chloro phenol and *p*-cresol. The aim of the study was to evaluate the influence of the type of phenolic substrates utilised by *T. cutaneum* R57 on the kinetics of the degradation processes described by a Haldane kinetic model. The effect of different initial concentrations on the biodegradation rate of resorcinol and 2,6-dinitrophenol is reported.

## Materials and methods

### Microbial cultivation

*T. cutaneum* R57 is registered in the National Bank of Industrial Microorganisms and Cell Cultures N2414/94, Bulgaria. It has previously been described for its ability to degrade phenol and other toxic aromatic compounds. The performed earlier analyses for pathogenicity and virulence of the studied strain showed strong negative results.

*T. cutaneum* R57 strain was grown in 50 mL liquid carbon-free yeast nitrogen base medium without amino acids medium (YNB w/o AA): 6.7 g L^−1^ supplemented with different concentrations of phenol, resorcinol, hydroquinone, *p*-cresol and *m*-nitrophenol, 2,6-dinitrophenol and *m*-chlorophenol. Each of these compounds was used as a sole carbon source in the biodegradation experiments.

The batch experiments were carried out on a New Brunswick rotary shaker at (28–30 °C) and 200 r min^−1^. Cell concentration was measured as optical density at λ = 610 nm with a Jenway 6306 spectrophotometer. The initial OD_610_ values in the experiments were adjusted to 0.135 ± 0.02.

### Analytical procedures

The degradation capacity of the yeast strain was evaluated based on the decrease in the concentration of aromatic compounds. The cell-free supernatants were analysed by high performance liquid chromatography (HPLC). HPLC was performed on a reverse phase C18 column (Licrosorb Rp18, PerkinElmer) with a methanol–water (50:50) mobile phase, using an ultraviolet detector set at 220 nm.

The data shown in the figures are mean values from at least three experiments.

### Mathematical model

The kinetic model describing the biomass growth and biodegradation of different aromatic compounds was presented as follows:(1) 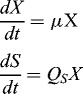
where *X* is biomass concentration; *S* is substrate concentration; 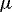 is specific growth rate and 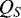 is specific degradation rate.

In this study, the specific degradation rate 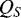 was described by a modified Haldane equation by replacing the specific growth rate to the rate of degradation, [23](2) 
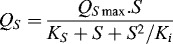
where 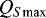 is maximum specific degradation rate; *K_s_* is saturation constant and *K_i_* is inhibition constant.

The parameter identification problem was reduced to minimisation of the following performance index:(3) 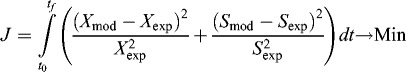
where *X*_mod_, *S*_mod_ and *X*_exp_ and *S*_exp_ are the model and experimental data for biomass and substrate concentrations, respectively; *t*_0_ is the initial process moment and *t_f_* is the final process moment indicating the time of degradation.

The unknown parameters were estimated using the optimisation procedure for direct search. Since the nonlinear optimisation procedure is strongly sensitive to the initial values and the variation intervals of the model parameters, the search for the values of the kinetic constants was constrained within boundaries predetermined on the basis of the process knowledge and experimental data. The coefficients of correlations *R* ^2^ were found to be more than 0.98, which indicated the model results agreed with the experimental data very well.

## Results and discussion

The relationship between the chemical structure and the levels of degradation of toxic chemical contaminants provides an approach for predicting and evaluating the effectiveness of microbial destructors in the development of biotechnological processes involved in cleaning the environment.

**Figure 1.**
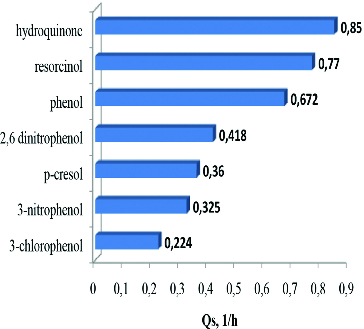
Maximum specific degradation rate for phenol and phenol derivatives by *T. cutaneum* R57.

In our study, biodegradation experiments were conducted with each of the tested compounds added to the carbon-free medium at a concentration of 0.2 g L^−1^. An exception was made for *m*-nitrophenol and *m*-chlorophenol because these compounds had a highly toxic effect on *T. cutaneum* R57 cells and complete degradation was observed only at a concentration of 0.1 g L^−1^. The maximum values of the rate of degradation (*Q_s_*) for the different compounds were compared. The obtained data indicated that the hydroxylated phenols were most rapidly degraded. The most intensive degradation was that of hydroquinone followed by resorcinol (Figure 1).

The results clearly demonstrated the dependence of the degradation rate on the presence of various substitution groups in the structure of the utilised compounds. This effect is directly linked to their inhibitory effect on the strain's cells. The results showed that the maximum degradation rates varied greatly (Figure 1). The most toxic chlorinated phenol was characterised with the lowest *Q_s_* value, while the highest *Q_s_* value was observed for less toxic hydroxylated phenols.

Similar conclusions have been made in a study on the aerobic biodegradability of phenol, resorcinol and 5**-**methylresorcinol and their different two-component mixtures using activated sludge sampled from a wastewater treatment plant. Nonlinear regression analysis used for determination of the kinetic parameters and short-term oxygen demands showed that they were functions of the compound undergoing biodegradation and the composition of the microbial community performing the degradation.[24]

It was interesting to observe that the order of the tested compounds was mostly in parallel with the decreasing of the *K_i_* (constant of inhibition) values.[14] An exception was found for 2,6-dinitrophenol characterised with a low *K_i_* value (0.13 g L^−1^), which might be probably due to the presence of two nitro groups linked to the benzene nucleus of this phenolic derivative. The effect of more than one substitution group on the enzyme activity and related degradation processes has long been known and described.[25] Despite the low value of the inhibitory constant, indicating strong inhibition of the culture development, the quite low value of the saturation constant (*K_s_* = 0.11 g L^−1^) should be noted. This fact demonstrated the ability of the enzyme apparatus of the *T. cutaneum* R57 strain to overcome the strong general inhibition and to metabolise effectively 2,6-dinitrophenol (Table 1). This feature may provide an explanation for the seemingly atypical effect of the substrate structure with two nitro groups on the degradation rate, e.g. the higher degradation rate observed for 2,6-dinitrophenol compared to the degradation rates established in experiments with mono phenol derivatives, e.g. *m*-nitrophenol and *p*-cresol (Figure 1).

**Table 1.** Kinetic parameters of resorcinol and 2,6-dinitrophenol degradation by *Trichosporon cutaneum* R57.

*S*_0_ (g L^−1^)	*T* (h)	*K_s_* (g L^−1^)	*K_i_* (g L^−1^)	*Q_s_* (h^−1^)
2,6-Dinitrophenol				
0.2	4	0.11	0.13	0.418
0.4	9	0.11	0.13	0.252
0.7	18	0.11	0.13	0.0912
				
Resorcinol				
0.2	6	0.22	0.58	0.77
0.6	10	0.22	0.58	0.644
0.8	15	0.22	0.58	0.28

The biodegradation capacity strongly depended on the concentration of the tested compounds as well. This has been addressed in only a limited number of reports. The impact of these important factors was investigated by comparative analysis of the kinetics of microbial degradation processes. The nature of the relationship of the rate of biodegradation regarding the concentration showed inhibition behaviour depending on the type of substrate.[26] Kinetic studies of the biodegradation of phenol in wastewaters have indicated that phenol concentration was the only major factor limiting the growth of immobilised cells of *T. cutaneum*. It was shown that the substrate concentration significantly influenced the values of growth and degradation rates.[18]

**Figure 2.**
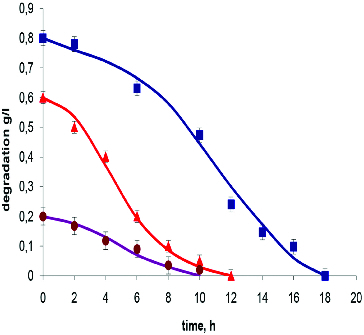
Experimental data and model output for batch degradation of resorcinol by *T. cutaneum* R57. Symbols indicate the initial concentration of resorcinol: 0.2 g L^−1^ (•); 0.6 g L^−1^ (▴) and 0.8 g L^−1^ (▪).

According to many authors, the Haldane equation can adequately express the dependence between substrate concentration *S*, *K_i_* and *Q_s_*.[12] The effect of substrate concentrations in the experiments for degradation of two of the investigated compounds: resorcinol (*K_i_* = 0.58 g L^−1^, low inhibitory effect) and 2,6-dinitrophenol (*K_i_* = 0.13 g L^−1^, strong inhibitory effect), was investigated in the next series of our experiments. It was observed that the increasing of concentration of both compounds led to a longer time necessary for their complete utilisation as a result of the decrease of the degradation rate. The degradation kinetics of *T. cutaneum* R57 in the presence of different concentrations of resorcinol showed an expected decrease in the *Q_s_* values in parallel with the increased substrate concentration. The degradation curves are demonstrated in Figure 2. The related model parameters are shown in Table 1.

Similar results have been obtained in experiments in which an indigenous mixed microbial culture was used in the study of the degradation of *m*-cresol incorporated in the growth media in concentrations ranging from 100 to 900 mg L^−1^. A maximum specific degradation rate of 0.585 h^−1^ was observed at 200 mg L^−1^ *m*-cresol concentration in the medium. In the range of *m*-cresol concentrations used in the study, the specific growth rate of the culture and specific degradation rates were observed to follow substrate inhibition kinetics.[17] Reduction of the degradation rate of phenol with increasing the initial phenol concentration has been reported in experiments with *P. fluorescence* cultured in medium containing 100 –500 mg L^−1^ phenol.[19]

**Figure 3.**
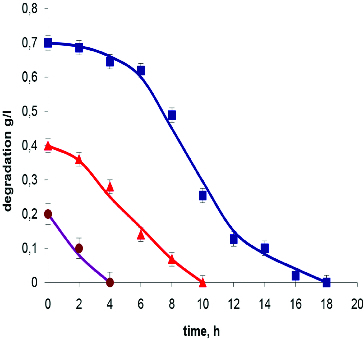
Experimental data and model output for batch degradation of 2,6-dinitrophenol by *T. cutaneum* R57. Symbols indicate the initial concentration of 2,6-dinitrophenol: 0.2 g L^−1^ (•); 0.4 g L^−1^ (▴); 0.7 g L^−1^ (▪).

The 2,6-dinitrophenol degradation curves are shown in Figure 3. The related kinetic parameters are presented in Table 1. A significant difference in the rate of degradation of various concentrations of this compound was observed.

The ratio of the *Q_s_* values found at a concentration of 0.2 versus 0.7 g L^−1^ was equal to 4.58. This was significantly higher than the ratio of these values found in the degradation of resorcinol – 2.75 (Table 1). This comparison demonstrated the specificity of the effects of high concentrations depending on the type of substrate, but also showed that the dependence of *Q_s_* was directly correlated with *K_i_* values. The data presented in Table 1 illustrated adequately the threshold substrate concentrations over which a reduction in the rate of degradation could be observed. This occurs when the initial concentration is comparable to the value of the corresponding *Ki*.

Some analyses of the influence of initial concentrations of phenol (the so-called rate of substrate consumption [*r_s_*_max_]), which is synonymous with the rate of degradation of the substrate (*Q_s_*_max_), make authors to believe that this parameter is most important in assessing the potential of microbial degradation.[19,27]

Kinetic studies on the degradation rate of seven phenolic compounds with different structure and toxicity by *T. cutaneum* R57 are important to reveal its degradation capacity and possibility for its application in biotechnological systems for purification of contaminated industrial waste. Development of models able to describe the characteristic changes in the process of degradation of phenolic compounds creates promising prospects for more effective management and control of technological schemes used in bioremediation of polluted soils and waters.

## Conclusions

The most intensive degradation of tested phenolic hydrocarbons by *T. cutaneum* R57 was observed for hydroxylated phenols as a sole carbon and energy source. The specific degradation rates (*Q_s_*) were described by a Haldane kinetic model. The order of *Q_s_* values for the tested compounds was mostly in parallel with the decreasing of *K_i_* (constant of inhibition) values. The effect of growing concentrations demonstrated increasing inhibitory effect, which directly correlates with the extended time necessary for complete degradation of both compounds. The results obtained in this study demonstrated that the rate of compound degradation is not strictly dependent on the values of the saturation constants expressing the ability of cellular enzymes to metabolise the relevant carbon substrates.
